# Change of Air and Baths in the Summer Diarrhœa of Children

**Published:** 1892-10

**Authors:** 


CHANGE OF AIR AND BATHS IN THE SUMMER DIARRHŒA OF CHILDREN.—(*Medical News*, June 18, 1892.)—Dr. Simon Baruch points out the advantages of change of air for relieving the disorders due to high temperatures, marked atmospheric humidity, and vitiated atmosphere. The floating hospitals and sea-side sanitariums conducted by the St. John's Guild, the Babies' Shelter and other charities furnish thousands of children in New York with the opportunity to escape for a day or longer, from the depressing influence of hot and impure air. Dr. Baruch says, "While for reasons already mentioned, a change is imperative in almost all cases of summer diarrhœa of the children residing in crowded tenement-houses, it is not so important in those cases whose environment is more favorable for home treatment. Indeed, the change from a comfortable home to a country hotel which is apt to be over-crowded, is not to be advised without careful reflection. It is not an infrequent occurrence to order a sick child away, when the symptoms become alarming, without time for preparation or due inquiry. The consequences are discomfort from immaturity of plans, great expense, disturbance of the family, and consequent anxiety and unhappiness for the parents and friends. Before deciding that the benefits which are likely to accrue to the little patients warrant these measures, it is imperative to be satisfied that we have exhausted all other methods of treatment, particularly sterilization of food and intestinal irrigation." Explicit directions are given as to bathing and the use of the wet pack. These measures ought to be more freely adopted.—*Inter. Med. Mag.*

